# Can Some Anticancer Drugs Be Repurposed to Treat Amyotrophic Lateral Sclerosis? A Brief Narrative Review

**DOI:** 10.3390/ijms25031751

**Published:** 2024-02-01

**Authors:** Rosa Luisa Potenza, Monica Armida, Patrizia Popoli

**Affiliations:** National Centre for Drug Research and Evaluation, Istituto Superiore di Sanità, 00161 Rome, Italy; monica.armida@iss.it (M.A.); patrizia.popoli@iss.it (P.P.)

**Keywords:** amyotrophic lateral sclerosis, anticancer drugs, repositioning

## Abstract

Amyotrophic lateral sclerosis (ALS) is a rare progressive motor neuron disease that, due to its high complexity, still lacks effective treatments. Development of a new drug is a highly costly and time-consuming process, and the repositioning of approved drugs can represent an efficient strategy to provide therapeutic opportunities. This is particularly true for rare diseases, which are characterised by small patient populations and therefore attract little commercial interest. Based on the overlap between the biological background of cancer and neurodegeneration, the repurposing of antineoplastic drugs for ALS has been suggested. The objective of this narrative review was to summarise the current experimental evidence on the use of approved anticancer drugs in ALS. Specifically, anticancer drugs belonging to different classes were found to act on mechanisms involved in the ALS pathogenesis, and some of them proved to exert beneficial effects in ALS models. However, additional studies are necessary to confirm the real therapeutic potential of anticancer drugs for repositioning in ALS treatment.

## 1. Introduction

Amyotrophic lateral sclerosis (ALS) is a rare neurodegenerative disease characterised by selective damage to upper and lower motor neurons, leading to death, usually as a consequence of respiratory failure, approximately 3–5 years after symptom onset [1,2]. The prevalence of ALS has been reported as between 4.1 and 8.4 per 100,000 and it is expected to grow, mainly due to the ageing population [3].

The pathophysiological mechanisms underlying the disease are still poorly understood [4]. As is the case with other neurodegenerative diseases, ALS genesis appears to be regulated from a complex interaction between individual genetic risks, aging and environmental factors [5]. More than 90% of ALS cases are sporadic, whereas about 5–10% are familial [1]. About 60% of familiar and 10% of sporadic ALS cases are due to pathogenic mutation in superoxide dismutase 1 (SOD1), TAR DNA-binding protein (TARDBP), fused in sarcoma (FUS) and chromosome 9 open reading frame 72 (C9orf72), the four most common ALS-associated genes [6].

Many ALS patients also show cognitive disturbances, extrapyramidal deficits and neuropathological findings, which reveal the multisystem nature of the disease [7]. Such a multifactorial nature partially explains why, in spite of intense basic research efforts, effective treatments remain elusive and ALS still represents an unmet medical need. Indeed, currently only two drugs, riluzole and edaravone, have currently received marketing authorisation for ALS treatment; moreover, their efficacy is rather limited [8].

The discovery of a new drug is a highly costly and time-consuming process, and the propensity of pharmaceutical companies to allocate their resources depends on the commercial potential of the future drug. Thus, in the case of rare diseases, the interest of private industry is often limited. The repositioning of drugs already approved for alternative indications could represent an efficient strategy to provide therapeutic opportunities for orphan diseases [9]. The advantages of this approach vs. de novo drug development are obvious. Indeed, for approved drugs, pharmacodynamics, pharmacokinetics and safety in the clinical setting are already established, making the research process quicker and less costly, subsequently allowing their rapid implementation into new medical applications. Repositioning may typically be considered when a drug acts on a pathogenetic mechanism that is shared by different diseases. From a more complex point of view, repositioning may rely on the “promiscuous” nature of the drugs, as they often interact with multiple targets, each of which may have a role in the pathogenesis of different diseases [10].

Some evidence has highlighted the existence of intriguing relationships between neurodegeneration and cancer [11,12]. In particular, despite the opposite hallmarks of the two conditions (excessive cell proliferation vs. cell loss), it has been suggested that some anticancer drugs might be repurposed for the treatment of neurodegenerative diseases, including ALS [13,14]. In agreement, we proposed in a previous article that fenretinide, an analogue of retinol endowed with antineoplastic activity, could additionally be considered as well for the treatment of ALS and other neurological disorders [15].

As far as ALS is specifically concerned, its possible interconnections with cancer have been explored by several studies supporting mutual links between these two age-related diseases [16]. Indeed, microarray analysis of ALS patient samples showed that candidate genes for ALS biomarkers are related to cancer development [17,18,19]. Other studies revealed common signalling pathways between ALS and cancer, such as Scr/c–abl, which was found to be overactivated during both cancer and ALS progression [20], and the P38 mitogen-activated protein kinase (p38MAPK) pathway [21], whose inhibition rescued the axonal transport defects in ALS mice [22].

Epidemiological studies on the possible association between cancer and ALS have reported discordant results. Fang et al. [23] reported no overall association of cancer and risk of ALS, while more cases of some specific tumours (specifically, prostate and brain) were diagnosed in ALS patients. Freedman et al. [24] reported that ALS mortality was not associated with total cancers, while the risk of ALS death was found to be increased or decreased when specific tumours were considered. Moreover, a longitudinal study showed a reduced overall risk of cancer, but an increased risk for salivary and testicular cancer, in ALS patients [25].

Such discrepancies may well be explained by the fact that speaking in terms of “cancer” as though it were a single disease can be misleading, since even cancers with the same histological origin can dramatically differ from one another in terms of clinical, prognostic and therapeutic issues according to their specific molecular profiles. Similarly, “anticancer drugs” is an umbrella term including very different molecules (and related mechanisms of action) that should not be regarded as a whole.

In the present review, we examine anticancer drugs belonging to different classes and discuss the mechanisms underlying their possible therapeutic roles on ALS. A detailed literature search of PubMed was performed to identify publications (including clinical studies, animal studies and in vitro studies) reporting use of already approved anticancer drugs in ALS. Clinical.trial.gov and DrugBank databases were also interrogated.

## 2. Miscellaneous

Fenretinide (DrugBank Accession Number DB05076, N-(4-hydroxyphenyl)retinamide) is a semisynthetic derivative of all-trans retinoic acid produced in the USA in the 1960s and was first proposed as an anticancer treatment due to its significant antitumour activity and favourable toxicological profile [26]. The antitumour effect of fenretinide is very complex and includes different mechanisms of action [15].

Even if it has not yet been approved by the EMA, fenretinide obtained orphan designation by the European Commission for the treatment of primary malignant bone tumours [27] and cutaneous T-cell lymphoma [28].

Very recently, we demonstrated that low doses (10 mg/kg) of a new fenretinide formulation significantly attenuates the neurological phenotype and extends the survival of mice expressing the mutated form of human SOD1 protein (mSOD1^G93A^ ALS mice), even when administered after the onset of motor symptoms [29]. We also demonstrated that in cultured motoneurons the expression of ALS-linked SOD1 mutation resulted in mitochondrial dysfunction, which can be reversed by treatment with fenretinide. The ability of FEN to protect myotubes from “in vitro” mSOD1 toxicity could partially explain the attenuation of the progression of neurological symptoms observed in mSOD1^G93A^ mice chronically treated with the drug [29].

Our results extended the neuroprotective potential of this anticancer drug to ALS treatment already reported for other neurological diseases like multiple sclerosis and Alzheimer’s disease [15]. The neuroprotective effects of fenretinide occurred at much lower doses than those required for its antitumour activity. Indeed, high doses of fenretinide can activate acute responses leading to ROS increase and cell death [30], while, at subtoxic concentrations, the drug can stimulate an adaptive stress response [31].

## 3. Alkylating Agents

The platinum complexes have revolutionised cancer therapy and the majority of chemotherapic regimens routinely applied in the clinical setting are still platinum-based [32]. These inorganic compounds are classified as alkylating agents and are used in the treatment of different forms of cancer, including sarcomas, carcinomas, lymphomas and germ cell tumours [33].

Cisplatin (DrugBank Accession Number DB00515) was the first member of its class, which now also includes carboplatin and oxaliplatin. As with all the other alkylating agents, cisplatin prevents the cell from dividing by adding an alkyl group to the DNA. However, only a small fraction of the administered dose reacts with the DNA to induce cytotoxicity, whereas the largest amount binds cellular proteins, thereby influencing other potential targets [34]. Calderone et al. showed that cisplatin selectively binds to His-19 residue located on the surface of the bovine SOD protein Cu/Zn superoxide dismutase (SOD1) [35]. SOD1 is an antioxidant enzyme that catalyses the dismutation of superoxide radicals; approximately 20% of familial ALS (FALS) cases are due to mutations in the SOD1 gene and, albeit at a very low frequency, SOD1 mutations are also observed in the sporadic form of the disease (SALS) [36]. Furthermore, not only the mutation but also the aggregation of the wild type SOD1 protein may play a role in modulating disease initiation [37]. In 2012, Banci and colleagues showed that cisplatin also interacts with the human form of SOD1, binding two cysteines (Cys6 and Cys111) onto the protein surface [38]. Cys6 and Cys111 residues were implicated in the aberrant aggregation of the mutated form of SOD1 [39], which are deemed to be essential in inducing endoplasmic reticulum (ER) stress related to SOD1 protein misfolding in ALS [40]. The potential use of cisplatin in the treatment of ALS was thus proposed [41].

Carboplatin (DrugBank Accession Number DB00958) is another platinum-based drug already approved to treat different forms of cancer. Due to its hydrophilic nature, carboplatin is longer retained longer within brain tissue; interestingly, it was found to be highly effective against glioblastoma while being nontoxic to normal brain tissue [42]. In breast cancer cells, carboplatin induced the expression of the omega class of cytosolic glutathione S-transferase (GSTO1) [43], an enzyme that is significantly reduced in peripheral blood mononuclear cells and in the spinal cord from ALS patients [44]. The glutathione S-transferase omega 1 (GSTO1) and 2 (GstO2, the Drosophila homolog of human GSTO1) were found to be involved in the oxidative damage underlying the pathogenesis of neurodegenerative diseases [45]. The overexpression of GSTO was shown to reduce the citoplasmatic accumulation of two proteins whose abnormal aggregations are characteristics of ALS and frontotemporal dementia [46], namely, the fused in sarcoma (FUS) DNA/RNA-binding protein and the Transactive response DNA-binding protein-43 (TDP-43) [47]. Specifically, Cha et al. showed that FUS neurotoxicity is sustained by impaired protein solubility induced by glutathionylation and that the overexpression of glutathione transferase omega 2 (GstO2) reduces abnormal protein aggregates in both TDP43 and FUS transgenic *Drosophila*, thus highlighting the therapeutic potential of carboplatin in ALS. Indeed, the drug rescued the mitochondrial disfunction and dose-dependently reduced locomotor and eye deficits in the FUS-ALS fly model [48].

## 4. Antimetabolites

Antimetabolites are nucleoside analogues interfering or competing with nucleoside triphosphates in the synthesis of DNA (antimitotic) or RNA or both. The fluoropyrimidine 5-fluorouracil (5-FU) (DrugBank Accession Number DB00544) is a pyrimidine analogue used as a palliative cancer treatment or to treat basal cell carcinomas. Besides its antimitotic effect, 5-FU can also induce striking alterations in RNA metabolism, splicing and post-transcriptional modification [49], suggesting the possible occurrence of several off-target effects. In a preclinical study designed to evaluate stem cell mobilisation in the murine ALS model, Rando et al. administered 5-FU as a negative control. As expected for an anticancer drug, the 5-FU administration induced a reduction of cellular component with a rapid turnover, as blood cells, an effect fully recovered after two weeks of repeated treatment. Surprisingly, however, when chronically administered in the pre-symptomatic phase, 5-FU also delayed the disease onset, improved the motor performance and increased the lifespan of ALS-treated animals, while it did not exert major effects on the myogenic, apoptotic or autophagic markers commonly elevated in mSOD1^G93A^ muscles [50]. Although no mechanistic data were provided by the above study, 5-FU has been reported to reduce tryptophan-induced SOD1 aggregation in cells [51]. This can be very relevant to the possible therapeutic effects of 5FU on human ALS, since tryptophan residue at position 32 has a critical influence on human SOD1 toxicity to motor neurons [52].

## 5. Hormone Antagonists

Tamoxifen (DrugBank Accession NumberDB00675) is a selective estrogen receptor modulator with both estrogenic and anti-estrogenic effects. In breast tissue, tamoxifen exerts anti-estrogenic and antitumour effects by blocking estrogens from entering cancer cells and thus reducing or eliminating the cells’ ability to grow and spread [53]. It is generally used to treat breast cancer in men and women and as a prophylactic treatment against breast cancer in women. Besides its antitumour activity, tamoxifen also showed neuroprotective effects in some preclinical models of neurological disease [54]. In experimental brain injury, the drug reduced neuroinflammation through TLR4/NF-kappaB pathways [55], while in a murine model of spinal cord injury it reduced microglia activation and the apoptotic death of neural cells [54,56]. Interestingly, in mice overexpressing TDP-43 DNA/RNA-binding protein (identified as the major component of the cytoplasmic inclusions in frontotemporal dementia and ALS), tamoxifen treatment was associated with an improvements in motor functions. The behavioural effect was accompanied by a reduction in the neuronal loss and TDP-43 inclusion in the forebrain of mice. Furthermore, in this murine model, tamoxifen also increased MTOR-dependent autophagy through AKT/PKB inhibition [56]. On the basis of the above results, a placebo-controlled randomised clinical trial was conducted in ALS patients without mutations in superoxide dismutase-1 (SOD1) or fused in sarcoma (FUS) genes [57]. Tamoxifen only modestly attenuated disease progression without exerting any significant effect on the primary clinical endpoint (time to death or dependence on mechanical ventilation, and tracheostomy with continuous mechanical ventilation and noninvasive ventilation for more than 12 h per day). This study must, however, be considered inconclusive; due to the extremely limited sample size (10 patients on tamoxifen and 8 on placebo), it was dramatically underpowered to detect a statistical significance in the clinical endpoint. Considering both the preclinical evidence and the inverse correlation between tamoxifen treatments and ALS risk reported in a population-based case–control study of >10,000 US cases [58], the potential therapeutic role of tamoxifen on human ALS seems worthy of further investigation in larger clinical studies.

## 6. Protein Kinase (PK) Inhibitors

Protein kinase (PK) inhibitors are a large group of antineoplastic agents that exert antiproliferative and cytotoxic effects through the inhibition of different pro-survival protein kinase activity. These drugs are generally categorised according to the amino acid that they phosphorylate and, among these, the tyrosine kinase receptor inhibitors remain the best characterised.

The c-Src and c-Abl tyrosine kinases are normally activated only in response to external signals such as molecules released following brain injury (adenosine, cytokines and ROS), and their activation was found abnormally elevated in neurodegenerative diseases [59]. Specifically, Src overactivation can trigger neuronal entry into aberrant cell cycles and induces post-mitotic death [13], whereas c-Abl mainly acts through mechanisms such as neuroinflammation and oxidative stress [60].

A significant increase in c-Abl mRNA was detected in the motor neurons of patients with a sporadic form of ALS [61] and in the spinal cord of transgenic ALS mice mSOD1^G93A^ as compared with WT littermates [62]. In mSOD1^G93A^ mice, the increased expression of c-Abl protein was accompanied by an increase in its active phosphorylated form, thus suggesting that c-Abl could represent a potential therapeutic target for ALS.

Imatinib (DrugBank Accession Number DB00619, Gleevec—STI571), an inhibitor of the oncogenic Bcr-Abelson (Bcr–Abl) which arises from a chromosomal rearrangement (Philadelpha chromosome) in acute lymphoblastic leukaemia, was the first-in-class compound to receive regulatory approval [63] and was then followed by many others within the next 10 years. The effectiveness of imatinib in preventing neuronal death was subsequently explored in several diseases affecting the central nervous system, including Alzheimer’s disease, multiple sclerosis, Parkinson’s disease and spinal cord injury [64]. As for ALS, imatinib was the only kinase inhibitor in the group that decreased mutant SOD1 protein levels. In 2015, Rojas et al. showed that c-Abl activation induced by oxidative stress can be counteracted by imatinib, and that the drug protected motoneurons from mSOD1 astrocyte-mediated toxicity [65]. Interestingly, one ALS patient treated for 7 years with imatinib for chronic myeloid leukaemia manifested a clear worsening of their ALS symptoms shortly after TKIs were withdrawn [20]. Furthermore, imatinib also inhibits the PDGFA receptor tyrosine protein kinases, thus potentially affecting multiple ALS targets [66].

The potential role of c-Abl inhibition in ALS is strengthened by the finding that another c-Abl inhibitor, dasatinib (DrugBank Accession Number DB01254), was able to attenuate motoneuron loss, delay disease progression and extend the survival of mSOD1^G93A^ ALS mice. Administration of dasatinib also induced a dose-dependent reduction in both c-Abl phosphorylation and caspase-3 activation, thus suggesting that a suppression of apoptotic cell death of motor neurons played a role in the neuroprotective effects of the drug [62].

A phenotypic-based drug screening carried on induced pluripotent stem cells (iPSCs) from sporadic ALS patients demonstrated that more than half of the screened hits targeted the Src/c-Abl signalling pathway. The authors provide evidence that the selected Src/c-Abl kinases inhibitor bosutinib (DrugBank Accession Number DB06616, which is approved for chronic myelogenous leukaemia) promoted autophagy and rescued ALS patient motor neurons from degeneration [67], whereas in an innovative 3D model of human ALS motor unit its co-application significantly increased the beneficial effects of rapamycin towards muscle contraction deficit [68]. The neuroprotective effects of bosutinib were confirmed in vivo in the mSOD1 mice model. When administered intraperitoneally (5 mg/kg per day) to ALS mice, the drug slightly delayed disease onset and extended survival. Such effects were accompanied by a decrease in misfolded SOD1 protein and motor neuron death in the spinal cords of treated mice [67]). The addition of a good brain penetration after systemic administration [69] further enhances the interest of the potential role of bosutinib in the treatment of neurological diseases. On these bases, a phase I clinical trial was approved by the Japanese Pharmaceuticals and Medical Devices Agency (Trial registration number UMIN000036295) to evaluate bosutinib for ALS patients (iDReAM) [70]. Although the study was mainly focused on the safety and tolerability of the drug and its efficacy only represented an exploratory endpoint, >50% of bosutinib-treated patients appeared to maintain clinical stability [71]. Considering the very limited number of patients enrolled and the short period of treatment, however, further clinical trials will be required to support the possible use of bosutinib in ALS patients.

Masitinib (DrugBank Accession Number DB11526) is the first anticancer therapy approved in veterinary medicine for the treatment of unresectable canine mast cell tumours. Masitinib selectively inhibits the c-KIT receptor, reducing the adverse effects of an overall TK inhibition [72]. The c-KIT receptor in humans is mainly involved in cancer and inflammation and masitinib was found to inhibit neuroinflammation-related symptoms by acting on mast cells and microglia [73]. In agreement, masitinib treatment was found beneficial in multiple sclerosis [73] and Alzheimer’s disease [74], two neurodegenerative diseases in which, as is the case with ALS, neuroinflammation plays a major role. Trias et al. firstly reported that oral masitinib decreased microgliosis and increased survival time of transgenic mSOD1-rats even when delivered after the onset of paralysis [75]. Subsequently, the same group demonstrated that the effects of masitinib in ALS also involved mast cells accumulated around degenerating motor axons and contributing to distal axonopathy and paralysis progression [76,77,78], thereby indicating that, besides microglia, mast cells represent a target of masatinib in ALS pathology. Accordingly, the drug was very recently reported to prevent mast cell infiltration and accumulation around spinal motor neurons of symptomatic mSOD1^G93A^ ALS mice [79]. As for ALS patients, a phase 2/3 randomised, double-blind placebo-controlled clinical trial showed that masitinib plus riluzole significantly reduced decline in the ALS Functional Rating Scale–Revised (ALSFRS-R) as compared to riluzole alone [80,81]. The European Medicines Agency (EMA) is now expected to issue a decision on the conditional approval of Alsitek (masitinib) as an add-on oral therapy for ALS.

## 7. Monoclonal Antibodies

Monoclonal antibody (mAb)-based immunotherapy is currently regarded as a crucial option in cancer treatment.

After its identification, the CD20 protein was found to be highly expressed on cancerous B cells in non-Hodgkin’s lymphoma, whereas it was not present on healthy immature B cells. Consequently, in the 90s, CD20 became the first target for mAb therapy and the anti-CD20 mAb rituximab (DrugBank Accession Number DB00073) was originally approved by the US FDA as a single agent to treat B-cell non-Hodgkin’s Lymphoma [82].

Rituximab and second-generation anti-CD20 are now used in the treatment of different malignant and non-malignant diseases, including neuroinflammatory conditions such as multiple sclerosis [83,84].

Unpublished data from Lichtenstein’s Lab suggested that reducing B cell counts by treating pre-symptomatic mSOD1^G93A^ mice with rituximab extended ALS mice survival. However, preclinical or clinical trials examining rituximab’s efficacy in ALS are still lacking [85].

## 8. Vinca Alkaloids

Vincristine (DrugBank Accession Number DB00541) is a vinca alkaloid isolated from Vinca Rosea and it is approved and marketed under several brand names for the treatment of acute leukaemia, malignant lymphoma, Hodgkin’s disease, acute erythraemia and acute panmyelosis.

The antitumour activity of vincristine is due primarily to the inhibition of mitosis by binding the β-subunit of tubulin heterodimers, leading to the arrest of division at metaphases and subsequently to cell death. Binding to the β-tubulin of axon microtubules leads to peripheral neuropathy, representing the major side effect of the long-term administration of vincristine [86]. However, at lower doses or in the presence of a higher clearance rate, vincristine binds tubulin in a reversible manner; its antitumour efficacy as well as its neurotoxicity are thus strictly dependent on the dose and the length of exposure [87].

Vincristine doses lower than those typically used in cancer chemotherapy have been proven to delay the onset of motor deficit and improve the lifespan of mSOD1^G93A^ ALS mice; these beneficial effects were associated with a reduction in microglial cell proliferation, which in ALS sustains neuroinflammation by the release of pro-inflammatory cytokines [88].

Due to its ability to increase platelet counts at low doses, vincristine is also used to treat patients with immune thrombocytopenia [89,90]. As ALS patients’ platelets were found to be more aggregated or grouped than controls [91,92], the effects of vincristine on platelet activation should also be taken into account when considering its possible use in ALS treatment.

## 9. Immunomodulating Agents

Thalidomide (Thalomid^®^ DrugBank Accession Number DB01041), a glutamic acid derivative, was first proposed as a sedative in the late 1950s and subsequently withdrawn in 1961 when it was shown to be teratogenic, causing severe birth defects (phocomelia) when given to pregnant women.

Several decades later, thalidomide was additionally found to exert immunosuppressive, anti-inflammatory and anti-angiogenic effects, suggesting a therapeutic use in inflammatory and malignant diseases. Indeed, in 1998, the Food and Drug Administration approved thalidomide as a therapy for erythema nodosum leprosum, and subsequently the drug was also approved for the treatment of multiple myeloma [93].

Mechanistically, one of the primary effects of thalidomide is the selective inhibition of TNF-α synthesis by activated monocytes and microglia [94]. Such a mechanism makes thalidomide potentially useful to mitigate neuroinflammatory-associated diseases [95].

In 2006, Kiaei et al. showed that the oral administration of thalidomide or its immunomodulatory imide drug analogue lenalidomide (Revlimid^®^, DrugBank Accession Number DB00480), used to treat multiple myeloma and anaemia in low- to intermediate-risk myelodysplastic syndrome, was able to modify the disease course and to extend the lifespan of mSOD1^G93A^ ALS mice. Such effects were associated with a reduction in TNF-α levels and motor neuron death [96]. A subsequent study showed clear protective effects of lenalidomide even if administered (orally) in already symptomatic mice [97].

Unfortunately, however, the available clinical evidence does not support the use of these compounds to treat ALS, as in a phase II clinical trial thalidomide not only failed to improve the ALS Functional Rating Scale (ALSFRS) and/or the pulmonary function of patients, but also induced several side effects [98].

## 10. Concluding Remarks

Building upon the consideration that neurodegeneration and cancer share some common mechanisms, it has been suggested that anticancer drugs might be repurposed for the treatment of neurodegenerative diseases [13,14]. Drug repurposing is particularly interesting for diseases that are at the same time rare and very serious, and for which no effective treatments are available. ALS represents a paradigmatic example of the above class.

**Figure 1:**
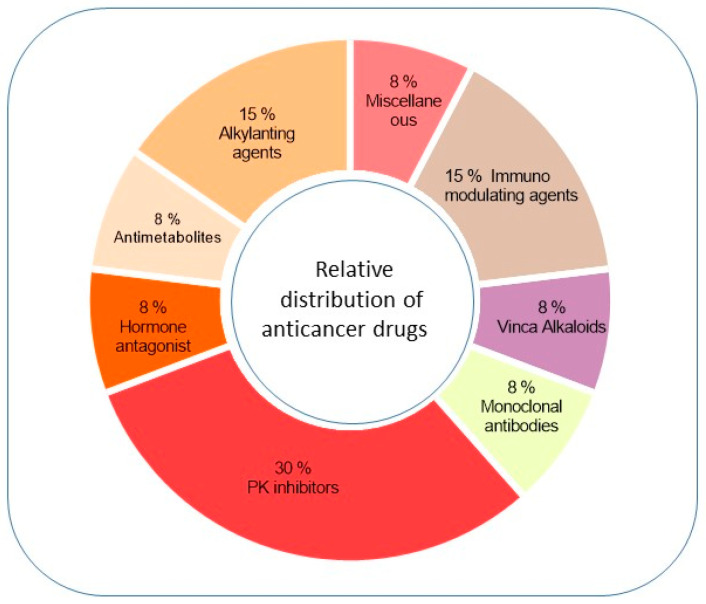
Relative distribution of considered anticancer drugs in different classes. The pie chart shows the percentage distribution into the different classes of antineoplastic drugs of the molecules considered here for a possible repositioning in ALS.

In the present review, we examined anticancer drugs belonging to different classes (Figure 1) and discussed the mechanisms underlying their possible therapeutic role in ALS (Table 1).

This seems at odds with the obvious observation that—since anticancer and anti-ALS treatments should operate in opposite directions (i.e., counteracting vs. promoting cell survival), thus having opposite effects—there is no way the same drugs could be used to treat both conditions.

An effective example arises from autophagy and apoptosis, which play a central role in both conditions. Anticancer therapies are designed to decrease uncontrolled cell proliferation by boosting apoptosis, whereas the inhibition of apoptosis and promotion of autophagy, whose failure leads to neurodegeneration and cell death, are attempted to treat neurodegenerative diseases [99]. However, depending on the administered dosage, the same drug can have opposite effects; a behaviour known as hormesis (namely, the phenomenon in which small doses of toxins and other stressors show stimulative effects [100]). Hormetic behaviour could explain the effectiveness of some anticancer drugs in ALS disease. For example, the anticancer drug fenretinide can act as a pro-apoptotic/pro-autophagic drug in a concentration-dependent manner [15]. Used at a sub-toxic dosage, chronic fenretinide administration was able to delay locomotor deficit progression in ALS mice, preserving muscle cells and motor neurons from mutant SOD1 toxicity [29]. Hormesis could also explain the effects elicited by the anticancer drug 5-FU in ALS mice [50], since it was reported to show hormetic reactions on cultured cancer cells [101]. Again, vincristine, used at doses lower than those normally employed as anticancer treatments, was found to delay disease progression in ALS mice [88].

Besides hormesis, another possible explanation for the apparent effectiveness of anticancer drugs in neurodegenerative conditions lies in their multitarget activities. This may be particularly relevant for a multifactorial disease like ALS, in which a traditional drug discovery approach (single target—single drug) may not be enough to fill the unmet need. Indeed, a multitarget pharmacology has been described for the majority of small molecule anticancer drugs [102]. Although such mechanisms are still poorly studied, their possible role in drug repositioning deserves further investigation.

Of interest within this review appears to be the polypharmacology of immunomodulatory imide drugs (thalidomide and its derivate lenalidomide), which exert their theratogenic and anticancer activity primarily by targeting the human cerebral protein with the Ion protease, Cereblon (CRBN) [103]. However, both thalidomide and its analogue lenalidomide were proven to down-regulate tumour necrosis factor-α (TNF-α) using a Cereblon-independent mechanism, suggesting a new therapeutic use for these drugs in disorders in which neuroinflammation exerts a pivotal role [95].

However interesting it may be, drug repositioning based on multitarget pharmacology is a very complex—and often unpredictable—approach. Indeed, to reduce the risk of unwanted effects in humans, almost every target should be identified and characterised. When repositioning is based on a pharmacological target that is never exploited within the clinical setting, further dose-response data may be required, and this might sometimes result in the need to return to the preclinical stages of regulatory approval.

Furthermore, proposing the use of cancer drugs as a treatment for neurological diseases requires a precise evaluation of cognitive function, as one of their major side effects affecting patients’ quality of life is a decline in learning, attention, executive functions, memory, multitasking and processing speed [104]. This condition, known as “chemobrain” or “chemofog”, on one hand confirms that anticancer drugs may influence central nervous system processes while also cautioning on the other hand about the possible neurotoxic effects of these drugs.

As a whole, the interesting evidence collected and discussed in this review seems to support the fascinating idea of repurposing anticancer drugs for ALS treatment. Hormesis and multitarget pharmacology could partially explain why drugs designed to block cell growth and survival should be used in neurodegenerative diseases.

The application of a network-based approach, which has already been successfully used for the repositioning of other pharmacological classes in ALS, has the potential to yield new knowledge [105]. Indeed, the evidence reported in this narrative review is mainly based on clinical and epidemiological evaluation in human studies and phenotypic change exerted by anticancer drugs in animal models. Combining a systematic computational approach based on the network medicine construct could lead to additional information on the potential of proposed repositioning [106].

However, the complexity of these phenomena, together with the poor knowledge of the CNS effects of anticancer drugs, dictates the need for a deeper mechanistic characterisation before considering this potential therapeutic option.

## Figures and Tables

**Table 1:** Anticancer drugs effects in ALS.

Pharmaceutical Family	Role in Cancer	Drug	Effects in ALS	References
**Miscellaneous**	Induction of apoptosis	Fenretinide	↓ oxidative damage in SOD1 motor neuron ↑ female survival time	[29]
**Alkylating Agents**	Antimitotic: interfere with DNA replication in cancer cells by adding an alkyl group to DNA	Cisplatin	↓ SOD1 protein misfolding linking SOD1 cysteine residues ↓ FUS aggregation and toxicity	[39,40]
		Carboplatin	↓ locomotor deficit FUS-ALS fly model	[48]
**Antimetabolites**	Antimitotic: interfere with DNA or RNA synthesis	5-fluorouracil	↓ mSOD1 aggregation toxicity delayed disease onset, ↑ motor performance ↑ survival time of ALS SOD1^G93A^ mice	[50,51]
**Hormone Antagonists**	Antiproliferative: inhibit the growth of sensitive cancer cell by antagonising hormone receptors	Tamoxifen	↓ Apoptosis ↓ microgliosis ↓ neuronal loss ↑ motor function	[56,57,58]
**Protein Kinase Inhibitors**	Antiproliferative and cytotoxic: inhibit pro-survival kinases in cancer cells	Imatinib	↓ oxidative stress in SOD1 motor neuron	[65]
		Dasatinib	↓ apoptotic motor neuron cell death ↓ motor deficit ↑ ALS mice survival time	[62]
		Bosutinib	↑ autophagy ↓ misfolded SOD1 protein ↓ spinal motor neuron death delayed disease onset ↑ survival of transgenic mice	[67,68,70,71]
		Masitinib	↓ microgliosis ↓ axonopathy ↓ mast cells infiltration ↑ survival time of ALS rats ↓ ALSFRS-R decline	[75,76,77,78,79,80,81]
**Monoclonal Antibodies**	Targeting CD20 protein on cancerous B cells	Rituximab	↓ B cell counts ↑ survival time of ALS SOD1^G93A^ mice	[85]
**Vinca Alkaloids**	Antimitotic; inhibit cancer cell division by inhibiting tubulin polymerisation	Vincristine	↓ gliosis in the spinal cord delayed disease onset in ALS mice	[88]
**Immunomodulating Agents**	Inhibition of angiongenesis and TNF-α production synthesis	Thalidomide/Lenalidomide	↑ body weight loss ↑ motor competence ↑ survival time of ALS SOD1^G93A^ mice	[96,97,98]

Superoxide dismutase 1: SOD1; mutant superoxide dismutase 1: mSOD1; fused in sarcoma: FUS; Tumour necrosis factor alpha: TNF-α; ALS Functional Rating scale–Revised: ALSFRS-R.

## Data Availability

Not applicable.
